# An Updated Protocol for High Throughput Plant Tissue Sectioning

**DOI:** 10.3389/fpls.2017.01721

**Published:** 2017-10-04

**Authors:** Jonathan A. Atkinson, Darren M. Wells

**Affiliations:** ^1^The Centre for Plant Integrative Biology, School of Biosciences, University of Nottingham, Nottingham, United Kingdom; ^2^BBSRC/Nottingham Wheat Research Centre, University of Nottingham, Nottingham, United Kingdom

**Keywords:** tissue sectioning, root anatomy, cross section, confocal microscopy, 3D printing

## Abstract

Quantification of the tissue and cellular structure of plant material is essential for the study of a variety of plant sciences applications. Currently, many methods for sectioning plant material are either low throughput or involve free-hand sectioning which requires a significant amount of practice. Here, we present an updated method to provide rapid and high-quality cross sections, primarily of root tissue but which can also be readily applied to other tissues such as leaves or stems. To increase the throughput of traditional agarose embedding and sectioning, custom designed 3D printed molds were utilized to embed 5–15 roots in a block for sectioning in a single cut. A single fluorescent stain in combination with laser scanning confocal microscopy was used to obtain high quality images of thick sections. The provided CAD files allow production of the embedding molds described here from a number of online 3D printing services. Although originally developed for roots, this method provides rapid, high quality cross sections of many plant tissue types, making it suitable for use in forward genetic screens for differences in specific cell structures or developmental changes. To demonstrate the utility of the technique, the two parent lines of the wheat (*Triticum aestivum*) Chinese Spring × Paragon doubled haploid mapping population were phenotyped for root anatomical differences. Significant differences in adventitious cross section area, stele area, xylem, phloem, metaxylem, and cortical cell file count were found.

## Introduction

Anatomical plant traits represent an important, yet relatively unexploited route for crop improvement, particularly in unfavorable or low-input conditions. For example, an increased number of root cortical aerenchyma (RCA) in maize has been shown to increase nitrogen acquisition in low nitrogen soils (Saengwilai et al., 2014). RCA in Maize have also been linked to reduced root respiration rate, leading to increased root growth allowing greater drought tolerance (Zhu et al., 2010). Other examples include increased metaxylem number, which have been linked to improved hydraulic conductivity under drought in high-yielding soybean lines (Prince et al., 2017), or decreased metaxylem size in wheat (*Triticum aestivum*), which has been linked to yield increases in dry environments (Richards and Passioura, 1989). In rice (*Oryza sativa L*.) leaf tissue, decreased photosynthesis and hydraulic conductance under drought has been lined to decreasing major vein thickness, rather than changes in leaf vein density (Tabassum et al., 2016). These traits are only quantifiable through histological sectioning techniques, which are often time consuming and difficult to conduct, limiting their use in forward genetic screens.

To date, histological sectioning often requires either sample fixation and storage, or hand sectioning of fresh material which can lead to a reduction of image quality, due to section thickness or sample damage (Lhotáková et al., 2008; Zelko et al., 2012). In cases where the plant tissue is fragile, such as thin roots, sectioning requires a high level of user skill to perform by hand (Zelko et al., 2012) or requires the time-consuming step of paraffin wax or resin embedding prior to sectioning using a microtome (Ruzin, 1999). In addition to this, the sample fixation and infiltration steps often cause softening or shrinking of tissues, leading to deformations in the sections taken. A comprehensive set of protocols for traditional sectioning techniques is available in Ruzin (1999). Other embedding media such as agarose has been successfully employed in several studies (e.g., Ron et al., 2013) and has the advantage that embedded specimens can be sectioned without fixation.

To some extent, advances in technology such as LAT (laser ablation tomography) are able to overcome these limitations while maintaining high throughput (Chimungu et al., 2014). LAT is also able to generate high resolution 3D images along a length of plant tissue, which is possible using traditional sectioning techniques but extremely time consuming (Wu et al., 2011). The main disadvantage to LAT is that the equipment necessary is bespoke, expensive, and currently unavailable to many research groups.

The work presented here aims to update and increase the throughput of existing methods (Lux et al., 2005; Zelko et al., 2012), while maintaining image clarity. Although this method was originally developed for root tissue, it is also applicable to other plant organs such as leaf and stem tissue.

## Methods

### Materials and Equipment

Calcofluor white solution (0.3 mg/ml)

Agarose (5% w/v)

Ethanol (70% v/v) or methanol (undiluted) for optional sample storage

Coverglass-bottomed cell chamber

3D printed embedding molds (see section “Embedding”)

Vibrating microtome (see section “Sectioning”)

Confocal laser scanning microscope (see section “Image collection”).

### Plant Material and Growth

Several plant species were used for analysis: wheat cv. Paragon, Chinese Spring and Savannah were grown in 2 l pots filled with potting compost (John Innes number 2) in a glasshouse; rice (*Oryza sativa*) was grown in hydroponic solution (nutrient concentrations as described in Murchie et al., 2005) in a controlled environment (CE) room at 28°C with a 12 h photoperiod and a light intensity at plant height of 400 μmol m^-2^ s^-1^; *Arabidopsis thaliana* seedlings were grown on agar plates containing half-strength Murashige and Skoog (MS) growth medium pH adjusted to 5.7 with KOH, in a CE room with 12 h photoperiod and a light intensity at plant height of 150 μmol m^-2^ s^-1^; *Thinopyrum ponticum* was grown in 2 l pots filled with potting compost (John Innes number 2) in a glasshouse.

### Sample Collection

**FIGURE 1:**
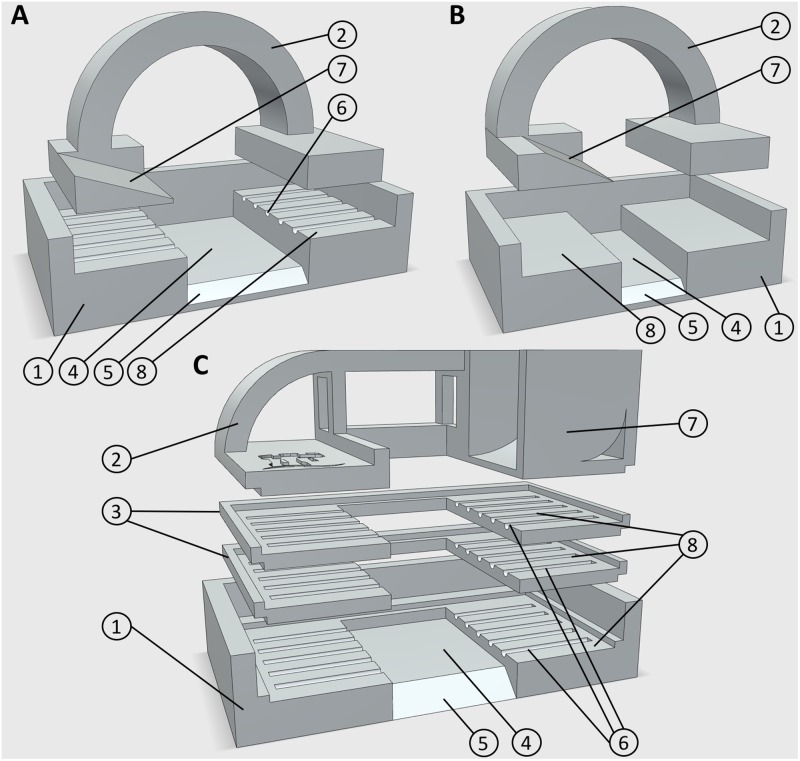
3D printed embedding molds. **(A)** Design 1, for use with five large diameter roots, or leaf samples. **(B)** Design 3, for use with smaller root samples. **(C)** Design 2, for use with up to 15 large diameter roots. Numbers represent components or design features. (1) Mold base (2) lid/root clamp (3) mid sections for additional sample layers (4) embedding media well (5) chamfer for block orientation (6) 0.5 mm guide groves for positioning root material (7) embedding media chute (8) sample recess. All designs are available in Supplementary Material in.STL file format.

Sections of wheat adventitious root were collected from soil at a depth of 5 cm and were carefully rinsed in water to remove excess soil. Five replicate plants were grown for each genotype, with four roots collected from each plant at anthesis. Rice adventitious and seminal roots were collected from 60 day old plants, 10 cm from the root tip and *Arabidopsis* primary roots were collected from 7 day old plants, 5 cm from the root tip. Direct contact with the samples at the point to be sectioned was avoided where possible, to prevent sample damage. All root samples collected were ∼3.5 cm in length. However, smaller ∼1.5 cm samples can be used with the third mold design (**Figure 1B**). Samples smaller than this can also be used if clipped into one side of the mold, but these may move during embedding and not be perpendicular to the blade for sectioning.

Wheat cv. Paragon leaf and stem samples (each ∼3.5 cm in length) were collected from plants grown in pots in the glasshouse as described above. Stems were collected from 2 cm below the ear at anthesis, or at the three leaf stage from seedlings. Leaf samples were collected from flag leaves during anthesis and longitudinally trimmed around the central vein to ∼6 mm width. Wider leaf sections can be used, but prevent multiple samples being sectioned simultaneously in a single block. *Thinopyrum ponticum* leaf samples were collected from the flag leaf of a flowering stem from ∼18 month old plants.

### Sample Storage

In most cases, samples were sectioned on the same day or the day following collection. Samples can also be stored long term in undiluted methanol (Neinhuis and Edelmann, 1996). Ethanol (70% v/v) can also be used on thick mature roots. It is also possible to store embedded samples (see below) for up to 2 days in water at 4°C.

### Embedding

Root samples were placed directly from growth or wash media into custom designed, 3D printed polylactic acid (PLA) molds in preparation for embedding (**Figures 1A–C**), unless being stored for sectioning at a later date. PLA is widely used in additive manufacturing for prototype creation and testing as it is relatively inexpensive. Most ‘prototyping plastics’ have suitable properties for this application, keeping the cost of each mold low. Other plastics commonly used in additive manufacturing such as high detailed resins or SLS nylon may also be used and give a smoother finish to the mold, but at a higher cost with no functional benefit. Stereolithography (^∗^.STL) files for all designs are provided in the Supplementary Material.

**FIGURE 2:**
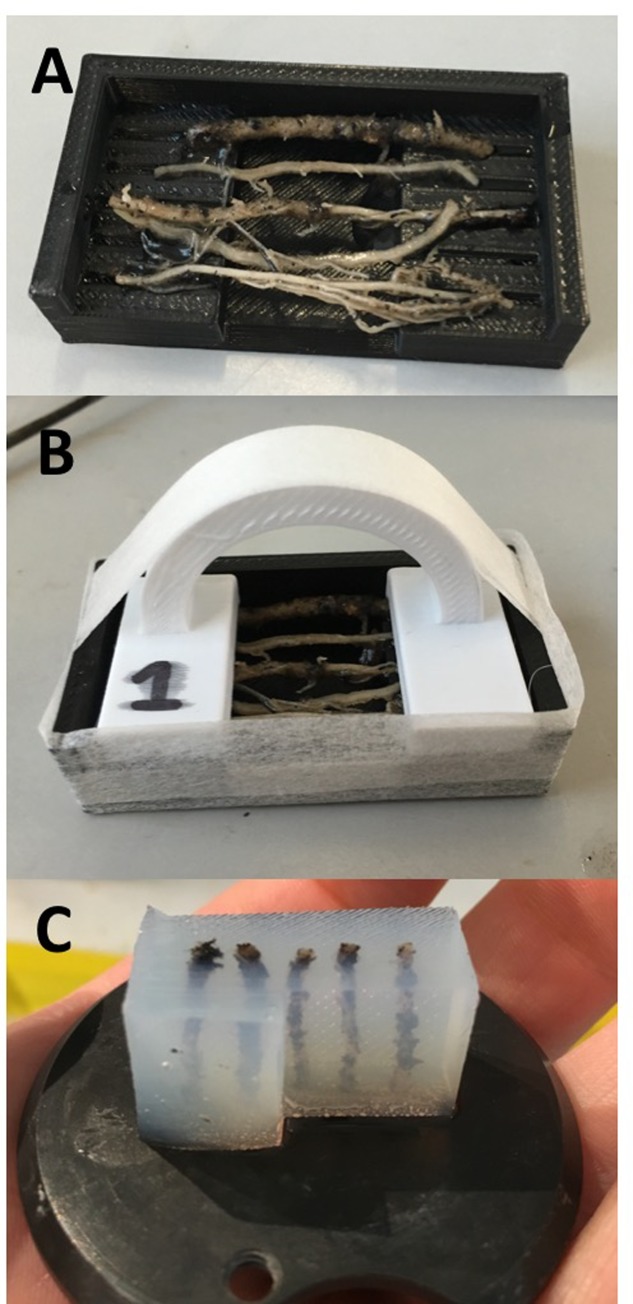
**(A)** Mold base containing five wheat adventitious roots. **(B)** Roots are clamped into place and the mold is sealed with pressure-sensitive tape ready for embedding. **(C)** Agarose block prepared for sectioning.

Currently, three mold designs have been developed: the first is for larger diameter roots (>400 μm) such as seminal and adventitious roots from wheat and rice, and can embed five roots in each block (**Figure 1A**); the second is also for large diameter roots, but can embed 5–15 roots in three layers **Figure 1C**); the third is for small diameter (100–400 μm) roots including lateral roots, or roots from species such as *Arabidopsis thaliana* (**Figure 1B**). Designs and CAD files can be found in Supplementary Material in STL format. Molds consist of a lower section with recesses for locating root samples, which suspends the roots over a well which is filled with agarose. Molds designed for larger diameter roots have grooved recesses to hold samples in place. Molds are assembled by fitting an upper section to clamp the samples prior to pouring the embedding medium. The second design has two additional middle sections allowing three layers of roots to be prepared (**Figure 1C**). When a large number of samples were collected (>50), populated molds were submerged in chilled water to avoid dehydration of roots prior to embedding. Each mold has a chamfer on the front edge, which allows orientation of the agarose block for use in individual sample identification (**Figure 2C**).

Cereal leaf and stem samples were embedded using the first mold design (**Figure 1A**). The second design is also suitable for embedding leaf and stem samples depending upon the species. Groves in the mold were ignored when placing these samples which were instead located to ensure a 4 mm gap between each sample, usually resulting in three leaf samples or four stem samples per mold.

5% (w/v) agarose (Sigma-Aldrich, Co. Ltd) was prepared in advance of placing samples into molds, using either a microwave or autoclave, and kept in an incubator at +55°C until use. Molds were sealed with pressure-sensitive tape (**Figure 2B**) and filled after leaving the agarose to cool to 39°C, close to the temperature of solidification. In species with finer roots (e.g., *A. thaliana*), less concentrated agarose (4%) can be used to avoid damaging peripheral cell files, although this may limit the thickness of cut sections. For leaf and stem samples, agarose was poured when removed from the incubator at +55°C; the higher temperature assists adhesion between the agarose and cuticular surfaces.

### Sectioning

A vibrating microtome [7000smz-2, Campden Instruments Ltd or VT1000s, Leica Microsystems (United Kingdom) Ltd] was used to achieve fast, reliable sections of between 80 and 250 μm. Agarose blocks were trimmed using a fresh double edge razor blade (Wilkinson Sword, United Kingdom), to remove excess sample material and agarose following removal from the molds. Samples were fixed to the vibratome sample mounting disks using cyanoacrylate adhesive (Loctite). Other vibratome models may use other sample mounts such as clamps. To ensure throughput was not effected by adhesive curing time, six mounting disks were used in rotation ensuring at least one sample was always ready for sectioning.

Vibratome settings used and section thickness varied between sample type and species. Typical settings for the two popular vibratome models used here are given in **Table 1**. Different settings may be required for other vibratome models.

**Table 1:** Vibrating microtome settings utilized for achieving sections with different sample types.

Sample type	Growth medium	Section thickness (μm)	Blade speed (mm/s)	Blade frequency (Hz)
Wheat adventitious root	Soil	200–250	1.75–2	70–80
	Hydroponics	100–250	1.50	80
Wheat seminal root	Soil	200–250	1.75	70
	Hydroponics	100–250	1.50	70
Rice adventitious root	Hydroponics	100–200	1.50	90
*Arabidopsis* primary root	Agar plate	80–100	1.50	70
Leaf (monocot)	–	100–140	1.25	60–80
Wheat stem	–	120–200	1.00	90


*Please note that a different vibrating microtome models and samples may require modification of these settings.*

### Staining

Root sections are removed from the vibratome bath and incubated in calcofluor white (Sigma-Aldrich, Co. Ltd) solution 0.3 mg/ml for 60 s, before being rinsed in deionised water. Sections were rapidly mounted using a drop of water onto a slide without a coverslip, or in a coverglass-bottomed cell chamber (Lab-Tek II Chambered Coverglass, Thermo-Fisher). The concentration and staining time requires optimisation for different sample types.

### Image Collection

Sections were observed using an Eclipse Ti CLSM confocal laser scanning microscope (Nikon Instruments). The microscope has three excitation lasers (405, 488, and 543 nm), three filter sets (450/35, 515/30, and 605/75), and four detectors. Images were collected using 10, 20, or 60x objectives depending upon the sample size. Larger samples were imaged in multiple overlapping positions (to facilitate assembly into a composite image).

**FIGURE 3:**
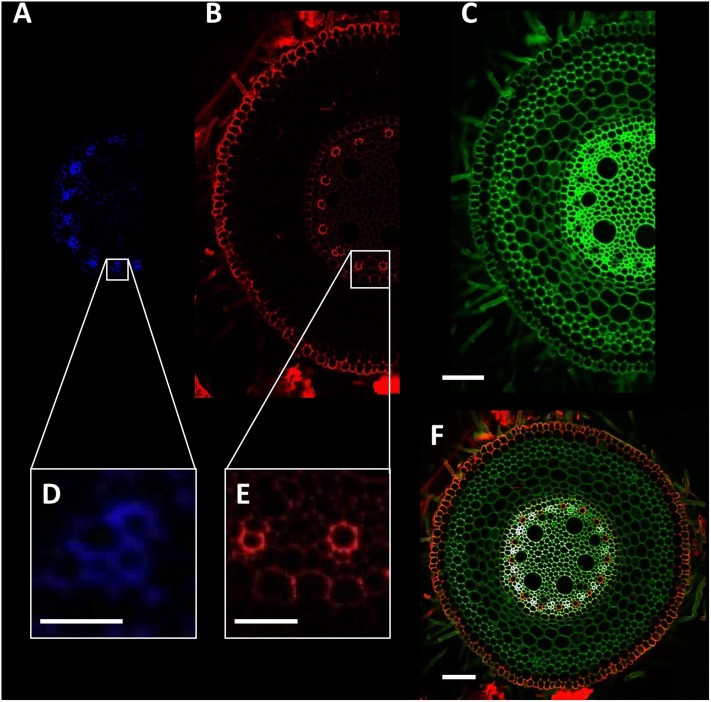
Detection of wheat root cellular features. **(A,D)** Phloem poles identified using excitation with the 408 nm laser and detection through a 450/35 filter. **(B)** Autofluorescence signal collected using the 488 nm laser and 605/75 filter. **(C)** Total cell wall image collected using 408 nm laser and 515/30 filter. **(E)** Xylem poles and Casparian strip formation. **(F)** Composite image of blue **(A)**, red **(B),** and green **(C)** channels. Scale bars **(A–C,F)** = 100 μm, **(D)** = 25 μm, **(E)** = 50 μm.

Detection of root anatomical features such as xylem, phloem, exodermis, endodermis, and Casparian band presence were achieved using a sequential combination of lasers and detectors to collect three image channels (**Figure 3** and **Table 2**). This was automated using the f-lambda feature, taking ∼30 s to capture all three images. Similar settings were utilized for leaf cross sections. For stem images, only the second and third image channels were used. For *Arabidopsis* roots, only the second channel was used.

**Table 2:** Confocal laser scanning microscope settings used to achieve multicolor images utilized for tissue detection and image capture.

Image channel	Laser (nm)	Filter	Gain	Pinhole size	Tissue
1	408	450/35 (blue)	65	Medium	Phloem
2	408	515/30 (green)	120	Small	All cell walls
3	488	605/75 (red)	100	Small	Xylem vessels, epidermis, and other secondary thickening


Individual channels were assembled into composite images using either the open source image software package Fiji (Schindelin et al., 2012), or in NIS-Elements Viewer (Nikon Instruments). Large objects imaged with a series of sub-images were stitched together using Autostitch (Brown and Lowe, 2007).

## Results

**FIGURE 4:**
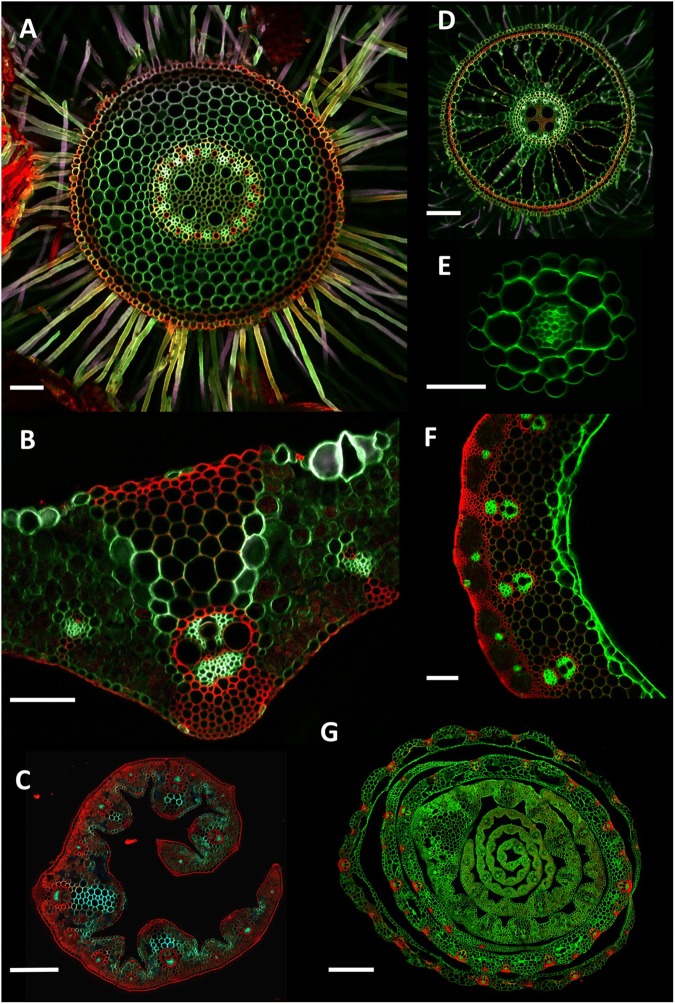
Example cross section images. **(A)** Wheat (*Triticum aestivum*) cv. Savannah mature adventitious root grown in soil. Collected 5 cm from stem, 250 μm thick. **(B)** Wheat cv. Paragon flag leaf central vein, 150 μm thick. **(C)** *Thinopyrum ponticum* leaf, 150 μm thick. **(D)** Rice (*Oryza sativa*) adventitious root grown hydroponically. Collected 10 cm above the root tip, 150 μm thick. **(E)** *Arabidopsis thaliana* primary root grown on an agar plate. Collected 5 cm from the root tip, 100 μm thick. **(F)** Wheat cv. Paragon stem. Sample collected 2 cm under ear at anthesis, 250 μm thick. **(G)** Wheat cv. Paragon seedling stem. Collected 4 cm above soil level, 200 μm thick. For **(C,G)**, due to the size of the samples, overlapping sub-images were collected and stitched together using Autostitch software. Scale bars **(A,B,D,F)** = 100 μm, **(E)** = 50 μm, **(C)** = 300 μm, **(G)** = 700 μm.

Here, we present a high throughput method for the collection and imaging of root and other plant tissue cross sections. If sampled the same day, ∼200 images of individual root samples can be achieved daily if the plants are being grown in artificial media such as hydroponics (**Figure 4B**). From soil, a rate of ∼160 roots per day was achieved using 35 of the 5 position molds (**Figure 1A**), when including sample collection (**Figure 4A**). A slightly lower rate of ∼140 images per day is possible with leaf sections as although sample collection and embedding is rapid, fewer leaves can be embedded in each block (**Figure 4D**). This represents a significant increase in throughput over traditional methods using resin or paraffin wax, where thick root samples can take several days to clear, infiltrate, and embed. It is also faster than free hand sectioning live tissue, as multiple samples are cut, stained and imaged together.

This throughput is achieved through a combination of 3D printed embedding molds and the use of a vibratome for fast, reliable thin sections. The use of confocal microscopy instead of standard epifluorescence microscopy allows higher contrast images to be taken with selective excitation of dyes and autofluorescence aiding identification of tissues.

By utilizing a mixture of autofluorescence and a single fast acting fluorescent stain (calcofluor white), it is possible to identify numerous cellular features such as xylem, phloem, exodermis, endodermis and Casparian band and aerenchyma formation in a single image (**Figures 3**, **4**), whilst maintaining high throughput.

The resulting root images are suitable for analysis using numerous existing software packages such as CellSet (Pound et al., 2012) PHIV-Rootcell (Lartaud et al., 2015) or RootScan (Burton et al., 2012). The multicolor images also allow for rapid manual measurement of specific cell types such as xylem, metaxylem, and phloem.

**FIGURE 5:**
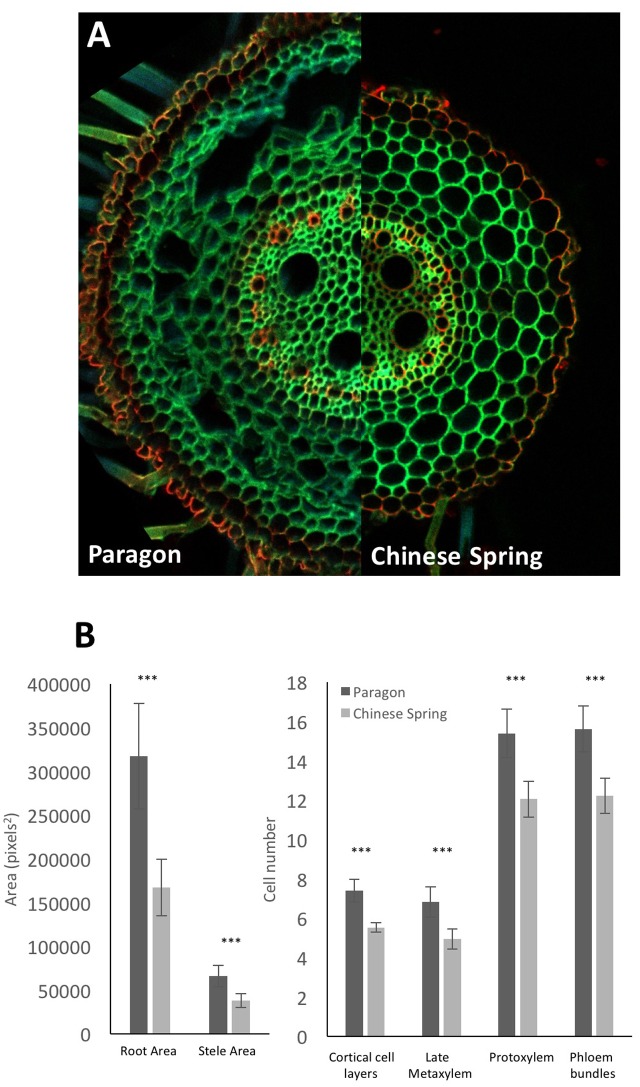
**(A)** Representative adventitious root cross sections of wheat cv. Paragon **(left)** and Chinese Spring **(right)**. **(B)** Quantified anatomical traits. Significant differences between cultivars were found using a two-tailed Student’s *t*-test for all quantified traits. ^∗∗∗^*p* > 0.001. Error bars = 2 × SE. Statistical tests conducted using Genstat 14th Edition (VSN International).

As an examplar experiment, adventitious roots of wheat cultivars Paragon and Chinese Spring were sectioned. The resulting images were manually analyzed for root cross sectional area, stele area, protoxylem cell count, late metaxylem cell count, phloem bundle count, and cortical cell layer number (**Figure 5**) with two-tailed Student’s *t*-tests conducted on the resulting data. Significant differences (*p* > 0.001) were found between the two cultivars for all quantified traits, with Paragon having significantly larger values in all cases.

## Discussion

Anatomical traits represent an important and yet relatively unexploited area of plant phenotyping, mainly due to the time-consuming nature of image and data collection. This method was developed with the aim of achieving high enough throughput to perform forward genetic screens for root cellular features, potentially requiring many 100s of images. Although already possible with methods such as LAT (Chimungu et al., 2014), the necessary equipment is bespoke and expensive and thus unavailable to most researchers. Here, we use a combination of 3D printing (universally available at low cost via online services) and confocal microscopy (available in many plant sciences research institutes) with a single, rapid florescent stain to increase the throughput of an already widely used method. Confocal microscopy provides the advantage of not requiring thin sections for image collection, increasing the speed at which sections can be cut and handled, as well as the detection of specific cell types via autofluorescence. A vibrating microtome can be used to achieve higher quality sections, but this is not strictly necessary. Although using a vibratome can reduce sectioning throughput, it can often reduce image capture time in blocks containing multiple samples, as the need to focus the objective between each sample is reduced or removed.

Root samples collected from different species grown in a variety of media including soil (both field and pots), hydroponics, growth pouches and agar plates were all successfully tested using this method, as well as leaf and stem tissue.

The main disadvantage of this method is that for best results, it requires the use of fresh tissue, which can cause logistical issues when sectioning large numbers of plants. Although time consuming, especially when sample collection is considered, it is possible to achieve ∼160 sections per day using fresh root tissue. To achieve this, significant preparation is required for efficient sample collection. Sectioning a large population of plants would require substantial planning with regards to timing. Fixation in undiluted methanol (Neinhuis and Edelmann, 1996) or 70–75% (v/v) ethanol (Meyer et al., 2009; Chimungu et al., 2014) is one possible solution, but can cause damage to peripheral cell layers in soft or thin root samples.

Another potential disadvantage is its inability to image specific cellular features, such as Casparian bands, in mature root tissue where the autofluorescence signal is often too strong. For this, more specific staining methods can be used such as those described in Zelko et al. (2012), although many of these methods require a significantly longer staining time, and would thus reduce throughput.

In the example experiment, significant differences in adventitious root traits between wheat cultivars Paragon and Chinese Spring have been found. These cultivars were selected as they are the progenitors of a doubled haploid mapping population (mapping data available from: http://www.cerealsdb.uk.net/cerealgenomics/CerealsDB/kasp_download.php), and thus any phenotypic differences have likely segregated in their progeny, making this mapping population a good candidate for analysis. With the increased throughput of this method, forward genetic analysis of this and other populations will be conducted in future studies.

## Author Contributions

JA designed the described method, components and collected all images and data utilized in this manuscript. DW provided guidance and help throughout this process as well as teaching and supervision. JA and DW contributed equally to writing the manuscript.

## Conflict of Interest Statement

The authors declare that the research was conducted in the absence of any commercial or financial relationships that could be construed as a potential conflict of interest.
