# Supplementary material for: An Updated Protocol for High Throughput Plant Tissue Sectioning
Source: Front Plant Sci. 2017 Oct 4;8:1721. doi: 10.3389/fpls.2017.01721 (PMC5632646; doi:10.3389/fpls.2017.01721)
Supplement: Supplementary file 4 [file Data_Sheet_1.DOCX]

Supplementary Material

An updated protocol for high throughput plant tissue sectioning

Jonathan A Atkinson* & Darren M Wells

*** Correspondence:** Jonathan Atkinson, jonathan.atkinson@nottingham.ac.uk

# Supplementary Material

Embedding mould designs are being released under CC BY 4.0 license, allowing free use and adaptation. Parts for each design are provided a .zip folder containing industry standard Stereolithography (*.STL) files for each component.

These files can be directly uploaded to a number of online printing services. Currently available international services include:

<https://www.3dhubs.com/>

<https://www.shapeways.com/>

<https://i.materialise.com/>

<https://www.sculpteo.com/>

Please note that this list is non-exhaustive.

Most ‘prototyping plastics’ (usually PLA) will have suitable properties for this application. High detail resins and SLS nylon are also suitable and give a finer finish, but usually at higher cost per component.

**List of files:**

Mould_A.zip: 5 positions for larger diameter roots (>400 μm). Consists of two components, a base and clamp which should both be printed for each mould.

Mould_B.zip: for smaller diameter roots (100-400 μm). Consists of two components, a base and clamp which should both be printed for each mould. We suggest printing at a minimum resolution of 100 μm to allow the clamp to hold samples in place.

Mould_C.zip: 15 positions for larger diameter roots (>400 μm). Consists of 3 components, a base, middle section (which should be printed twice per mould) and clamp. This mould should be printed with a maximum tolerance of 100 μm.
